# An Unusual Case of Drug-Induced Liver Injury Secondary to Nitrofurantoin Use

**DOI:** 10.7759/cureus.26882

**Published:** 2022-07-15

**Authors:** Stephen Wonnacott, Dhir Gala, Mili Shah, Diksha Kaul, Vikash Kumar

**Affiliations:** 1 Internal Medicine, American University of the Caribbean School of Medicine, Cupecoy, SXM; 2 Internal Medicine, The Brooklyn Hospital Center, New York, USA

**Keywords:** drug reaction, liver, transaminitis, drug-induced liver injury, nitrofurantoin

## Abstract

Nitrofurantoin is a commonly prescribed antibiotic for uncomplicated urinary tract infections. Despite a number of side effects, it is increasingly prescribed due to its low cost, high efficacy, and minimal antimicrobial resistance. One of the rare, however significant side effects of nitrofurantoin is idiosyncratic drug-induced liver toxicity. It commonly presents with abdominal pain and elevated liver enzymes. Interestingly, it can cause either an acute or a chronic hepatitis-like syndrome that can be severe and lead to liver failure or cirrhosis. We present a case of a healthy 24-year-old female who presented with epigastric abdominal pain, which was found to be drug-induced liver injury (DILI) secondary to her recent nitrofurantoin use.

## Introduction

Nitrofurantoin is an antibacterial drug of the nitrofuran family that is commonly prescribed for urinary tract infections (UTIs). Nitrofurantoin is prescribed both for short-term use against acute UTIs as well as long-term use as a prophylactic in patients that are prone to recurrent UTIs [1]. Nitrofurantoin was developed in the 1950s and was widely used until the 1970s, when better alternatives were developed. Nitrofurantoin started to gain popularity again in the late 2000s due to a rise in resistance to antimicrobials and is now considered a first-line treatment for uncomplicated lower UTIs [2]. The mechanism of action of nitrofurantoin is not well understood, but it is known to inhibit several bacterial enzymes by binding to bacterial ribosomes [3]. The recommended dosing of nitrofurantoin for acute UTI treatment is 50-100 mg four times per day for five to seven days, and the recommended dosing for long-term prophylaxis is 50-100 mg once per day [4]. One of the most severe adverse effects of the use of nitrofurantoin is drug-induced liver injury (DILI).

## Case presentation

A 24-year-old obese female urgent care worker with a past medical history of gastroesophageal reflux disease (GERD) presented to the emergency department complaining of epigastric pain that started a few hours before admission. She described the pain as burning with no radiation. It was associated with nausea and two episodes of non-bloody, non-bilious vomiting. She denied fever, chills, rash, joint pain, dysuria, frequency, urgency, recent trauma, herbal medicine use, or associated symptoms. Medication history included famotidine for GERD as needed and ibuprofen as needed for menstrual pain. Additionally, she was recently diagnosed with a urinary tract infection and had been taking nitrofurantoin (50 mg every six hours) for the past three days. Family history was significant for pancreatic cancer in her father. Her social history included occasional alcohol use (one to two drinks) about two to four times a month, last use was two weeks ago. She denied any tobacco use or drug use. 

On admission, the patient was afebrile with normal vital signs. The physical exam was unremarkable. An abdominal exam revealed a non-tender, non-distended abdomen with no signs of organomegaly. The blood tests were significant for direct bilirubin of 0.7 mg/dL, total bilirubin of 1.9 mg/dL, aspartate aminotransferase (AST) of >717 U/L, and alanine aminotransferase (ALT) of 476 U/L. Additionally, serum sodium, potassium, chloride, blood urea nitrogen (BUN), creatinine, glucose, albumin, international normalized rate (INR), and alkaline phosphatase levels were all within normal limits (Table 1). The patient's liver function tests done at the primary care physician's (PCP) office a week ago were normal. Toxicology labs were negative for acetylsalicylic acid (ASA), acetaminophen, and alcohol. Viral hepatitis workup was negative for hepatitis A, B, C, and E. Autoimmune workup, including antinuclear antibodies (ANA), anti-smooth muscle, anti-mitochondrial, liver kidney microsome type 1 (anti-LKM-1) antibodies, resulted in negative tests. 

**Table 1:** Laboratory results after discontinuation of nitrofurantoin

Parameters	Day 1	Day 2	Day 3	Normal values
Platelets	245,000	250,000	225,000	150,000-400,000/mm^3^
Total bilirubin	1.9	1.7	1.6	0.1-1.0 mg/dL
Direct bilirubin	0.7	0.8	0.6	0.0-0.3 mg/dL
Aspartate aminotransferase (AST)	>717	406	135	8-20 U/L
Alanine transaminase (ALT)	476	520	331	8-20 U/L
Alkaline phosphatase	120	129	104	20-70 U/L
Glucose	90	123	86	Fasting: 70-110 mg/dL; 2-h postprandial: <120 mg/dL
Albumin	4.5	4.1	3.7	3.5-5.5 g/dL
International normalized ratio (INR)	1.0	1.2	1.2	0.8-1.2 secs
Blood urea nitrogen (BUN)	11	5	6	7-18 mg/dL
Creatinine	0.8	0.8	0.7	0.6-1.2 mg/dL
Sodium	135	138	137	136-145 mEq/L
Potassium	5.8	3.7	3.8	3.5-5.0 mEq/L
Chloride	103	106	106	95-105 mEq/L
Aspirin (acetylsalicylic acid, ASA)	<5.0			5.0-20.0 mg/dL
Acetaminophen	<1			5.0-20.0mg/dL
Alcohol	<0.01			
Gammaglobulin	32			5-40 U/L

Ultrasound of the gallbladder showed stones within the gallbladder. There was no pericholecystic fluid, and the Murphy sign was negative, with no signs of cholecystitis. Nitrofurantoin was immediately discontinued, and after three days in the hospital, symptoms subsided. During the hospital course, the patient was monitored with neurological examinations to assess for signs of acute liver failure such as asterixis and encephalopathy. Repeat liver function tests showed enzyme values trending towards normal (Table 1). The patient was educated to follow up with PCP for repeat hepatic function test and chances of possible relapse with similar medications on discharge.

## Discussion

Nitrofurantoin is a commonly prescribed medication in primary care. Side effects include diarrhea, peripheral neuropathy, pulmonary fibrosis, interstitial pneumonitis, and hypersensitivity reactions. While most physicians are alert to the pulmonary complications of nitrofurantoin, the hepatic sequelae are not well described. DILI is a very rare but serious complication that can result from nitrofurantoin. It is the most common and lethal adverse drug reaction causing 25% of the fatal adverse reactions [5]. Acute liver injury from nitrofurantoin has a prevalence of ~0.3/100,000 prescriptions, while the prevalence of chronic nitrofurantoin liver injury is estimated to be one in 1500 [1]. There is a higher risk of developing DILI from taking nitrofurantoin for patients who are female, who have an increased age, who have reduced renal function, or who have an increased duration of treatment with the drug [5-7]. Human leukocyte antigens (HLA) types HLA-B8, HLA-DRw3, HLA-DR2 and HLA-DRw6 have all been associated with a higher risk of DILI; however, no statistical significance has been established as of yet [8-10].

DILI generally presents in two forms with nitrofurantoin, acute and chronic. The acute form can present within days or weeks from the start of treatment. It can present with fever, rash, eosinophilia, hepatomegaly, jaundice, abdominal pain, nausea, malaise, pulmonary signs, or anorexia [6,8]. The chronic form of nitrofurantoin-induced DILI generally presents after more than six months of prophylactic treatment. It generally progresses in an insidious manner presenting with many of the same symptoms minus the fever and rash [1,8]. The patient presenting with jaundice should be of great concern as it is associated with a 10% mortality rate [11].

Both forms of DILI generally present with elevated liver enzyme values; however, the chronic form is more associated with positive values for antinuclear antibodies, smooth muscle antibodies, lupus erythematosus (LE) cells, hypoalbuminemia, and hypergammaglobulinemia [8,12]. These elevated autoimmune markers have similarities to autoimmune hepatitis (AIH), so care must be taken to exclude other possible causes of AIH. With histology, it can also be difficult to distinguish between DILI and AIH, both presenting with inflammation, hepatitis, fibrosis, or even necrosis [5,12,13].

Most cases of acute DILI due to nitrofurantoin will resolve on their own after discontinuation of the drug but should be monitored in case chronic liver injury develops [1]. In chronic cases, liver biochemistry should return to normal after discontinued treatment; however, damage that has already occurred, such as cirrhosis, fibrosis, or necrosis, can require transplantation or lead to death [5]. However, in more severe cases, corticosteroids can be useful in improving the prognosis [5]. N-acetylcysteine (NAC) has also been shown to have some effectiveness at combating the oxidative damage caused by nitrofurantoin-induced DILI but may be limited by its own toxicity [14,15].

After developing nitrofurantoin-induced DILI, patients can become sensitized to the drug and have a high probability of relapse if the patient uses nitrofurantoin again [8,16]. Continued use of nitrofurantoin with elevated liver enzymes or subsequent rechallenge shows the most severe symptoms, including a majority of the DILI-related fatalities [8,16,17]. Due to an increased risk of hemolytic anemia and hepatotoxicity, nitrofurantoin is contraindicated in late pregnancy unless liver and blood values are monitored regularly [18].

## Conclusions

Nitrofurantoin is an uncommon cause of DILI associated with various clinical phenotypes, natural histories, and treatments. Patient demographic (age, gender, race) and laboratory features (serum ALT, bilirubin, INR) at DILI onset have been associated with the severity and outcomes of liver injury in patients with DILI. Early withdrawal of the medication nearly always results in rapid normalization of the liver biochemistry. Given the wide use of nitrofurantoin, it is important for clinicians to consider this adverse effect. Many studies suggest that medical interventions such as the use of NAC and corticosteroids may provide benefits to some patients, but additional studies are needed. Follow-up with hepatic function tests can be considered as a preventive measure after starting nitrofurantoin.
